# Eating behaviour profiles across the frontotemporal dementia spectrum

**DOI:** 10.1111/jnp.70045

**Published:** 2026-03-30

**Authors:** Yuki Sato, Hitomi Hayashi, Kazuo Kakinuma, Chifumi Iseki, Shoko Ota, Kazuto Katsuse, Shiho Matsubara, Nobuko Kawakami, Shigenori Kanno, Keisuke Morihara, Yoshiyuki Nishio, Kyoko Suzuki

**Affiliations:** ^1^ Tohoku University School of Medicine Sendai Japan; ^2^ Department of Behavioral Neurology and Cognitive Neuroscience Tohoku University Graduate School of Medicine Sendai Japan; ^3^ Department of Neurology Graduate School of Medicine, The University of Tokyo Tokyo Japan; ^4^ Department of Neurology and Stroke Medicine Graduate School of Medicine, Yokohama City University Yokohama Japan; ^5^ Department of Behavioural Neurology and Neuropsychiatry Osaka University United Graduate School of Child Development Suita Japan; ^6^ Center for Higher Brain Dysfunction Support Tohoku Medical and Pharmaceutical Hospital Sendai Japan

**Keywords:** disease progression, eating behaviour, feeding and eating disorders, frontotemporal dementia, neurobehavioural manifestations

## Abstract

Altered eating behaviours are a hallmark of behavioural variant frontotemporal dementia (bvFTD) but are less well characterised in progressive nonfluent aphasia (PNFA) and semantic dementia (SD). We investigated the frequency and onset of eating behaviour changes across the three subtypes. We retrospectively reviewed the data of 58 patients (14 bvFTD, 30 PNFA and 14 SD). The presence and onset of eating changes were assessed using the Neuropsychiatric Inventory and medical records. Eating behaviours were categorised into overeating, reduced food intake and food preference change. Primary outcomes were prevalence and incidence rates from the initial disease symptoms. A time‐to‐event analysis was used to compare the cumulative incidence of eating behaviour changes; cumulative incidence curves were estimated using the Kaplan–Meier method; and group differences were assessed using the log‐rank test. Although the prevalence of eating changes was the highest in bvFTD (85.7%), followed by PNFA (63.3%) and SD (57.1%), the differences were not statistically significant (*p* = .220). Incidence also did not differ significantly (*p* = .054). However, overeating was significantly more frequent in the bvFTD group than in the PNFA and SD groups (*p* = .011). Changes in appetite and eating behaviour are common across the frontotemporal dementia spectrum and are not limited to bvFTD. Although the overall prevalence is similar across subtypes, overeating is specifically observed in bvFTD, whereas reduced food intake and food preference changes occur non‐specifically.

## INTRODUCTION

Behavioural changes such as disinhibition and stereotypy are hallmarks of behavioural variant frontotemporal dementia (bvFTD) (Rascovsky et al., 2011), but they occur across the entire frontotemporal dementia spectrum (Jiskoot et al., 2023; Park et al., 2017; Rohrer & Warren, 2010; Rosen et al., 2006). Similarly, altered eating behaviours, including overeating and food preference changes, are core features of bvFTD (Cipriani et al., 2016; Rascovsky et al., 2011). However, these symptoms remain less well characterised in progressive nonfluent aphasia (PNFA) and semantic dementia (SD) (Gorno‐Tempini et al., 2011). Recent longitudinal studies have begun to characterise eating behaviour changes in these PPA variants (Foxe et al., 2022; van Langenhove et al., 2016). Longitudinal data regarding the interval between initial disease onset and the emergence of eating changes are crucial for early diagnosis and optimal intervention. Therefore, in this study, we aimed to compare the prevalence and cumulative incidence of eating behaviour changes across bvFTD, PNFA and SD within a single clinical cohort.

## METHODS

### Participants

We included consecutive patients who underwent initial clinical investigations for cognitive or behavioural symptoms at the Department of Behavioural Neurology and Cognitive Neuroscience between January 2017 and March 2024. Clinical diagnoses of bvFTD, PNFA or SD were established according to consensus diagnostic criteria (Gorno‐Tempini et al., 2011; Rascovsky et al., 2011). The final cohort comprised 58 patients: 14 with bvFTD (11 males), 30 with PNFA (18 males) and 14 with SD (8 males) (Table in Supporting Information).

### Assessment of appetite and eating changes

Appetite and eating changes were assessed using the Neuropsychiatric Inventory (NPI) (Cummings et al., 1994) administered upon initial evaluation, along with a retrospective review of outpatient medical records. We focused on the ‘appetite and eating change’ domain of the NPI. Specific symptoms were grouped into three categories: overeating (increased appetite, weight gain and binge eating); reduced food intake (loss of appetite and weight loss); and food preference changes (eating specific types of food excessively and stereotypic eating habits). The presence of these symptoms was treated as a binary variable (yes/no), without applying a frequency or severity threshold. This approach was chosen to uniformly integrate NPI data with retrospective medical records, which often lacked systematic severity or caregiver distress scores, and to evenly evaluate qualitatively different eating behaviours. The onset of these symptoms was determined based on caregiver reports. If the timing was unclear, the age at initial evaluation was used as the age of onset.

### Statistical analysis

Demographic and clinical characteristics were compared among the three diagnostic subgroups using one‐way analysis of variance for continuous variables and the chi‐squared test for categorical variables. The prevalence of eating behaviours (overeating, reduced food intake and food preference changes) was treated as binary data (yes or no) and compared using the chi‐squared test.

We performed a time‐to‐event analysis to compare the cumulative incidence of eating behaviour changes. The ‘event’ was defined as the age at the first occurrence of any appetite or eating change. ‘Time zero’ was defined as the onset of initial symptoms: the emergence of any behavioural or cognitive symptom consistent with consensus diagnostic criteria for bvFTD (Rascovsky et al., 2011) and the first observation of an objective linguistic disorder for aphasic variants (PNFA and SD). Cumulative incidence curves were estimated using the Kaplan–Meier method, and group differences were assessed using the log‐rank test. In the time‐to‐event analysis, the analysis time was defined as the ‘time since symptom onset’ (i.e. the age at the onset of eating changes minus the age at the onset of initial disease symptoms). For participants who did not develop eating changes, the censoring time was defined as the interval from the onset of initial symptoms to their last clinical assessment.

## RESULTS

**FIGURE 1:**
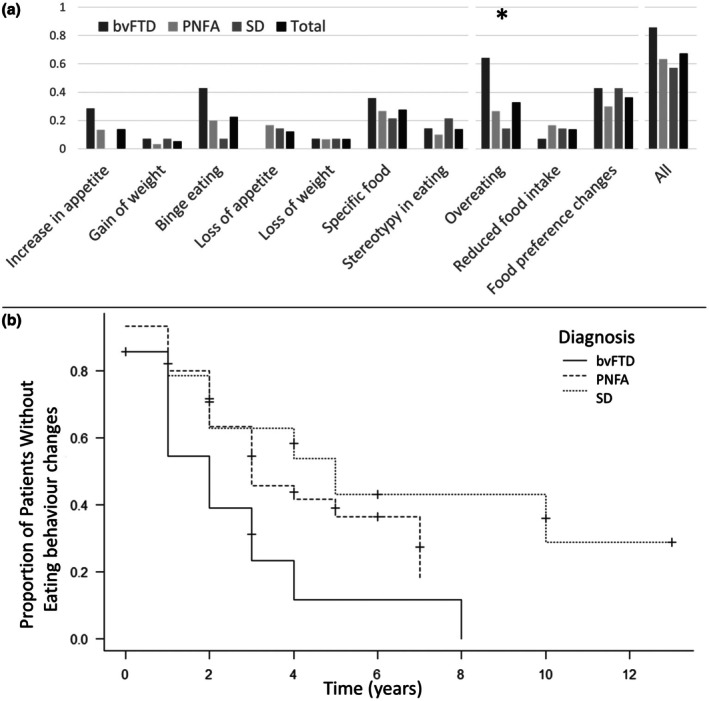
Prevalence and cumulative incidence of eating behaviour changes. (a) Prevalence of specific symptoms and categories across behavioural variant frontotemporal dementia (bvFTD), progressive nonfluent aphasia (PNFA) and semantic dementia (SD). The asterisk (*) indicates a significant group difference in overeating (*p* < .05). (b) Kaplan–Meier curves showing the cumulative incidence of any eating behaviour change from the onset of initial disease symptoms. ‘Time zero’ on the horizontal axis represents the onset of initial symptoms, and the analysis time was defined as the ‘time since symptom onset’ (i.e. the age at the onset of eating changes minus the age at the onset of initial disease symptoms). For participants who did not develop eating changes, the censoring time was defined as the interval from the onset of initial symptoms to their last clinical assessment.

Appetite and eating changes were present in 85.7% of patients with bvFTD, 63.3% of patients with PNFA and 57.1% of patients with SD (Table 1). No significant differences were observed among the groups (chi‐square test, *p* = .220). Regarding specific symptoms, the prevalence of overeating was significantly higher in the bvFTD group than in the PNFA and SD groups (chi‐squared test, *p* = .011). No significant group differences were observed for reduced food intake (chi‐squared test, *p* = .69) or food preference changes (chi‐squared test, *p* = .60; Figure 1a; Table 1). The symptom duration at the first assessment (age at first assessment minus age at onset of initial disease symptoms) showed no significant differences among the groups (ANOVA, *p* = .35).

**TABLE 1:** Demographic and clinical characteristics of participants.

Characteristic	bvFTD	PNFA	SD	Total	*p*‐value
*n*	14	30	14	58	
Sex (male)^a^	11 (78.6%)	18 (60.0%)	8 (57.1%)	37 (63.8%)	.411
Age at first assessment (years)^b^	65.4 ± 9.4	72.4 ± 5.9	66.4 ± 9.5	69.2 ± 8.3	.009
Changes in appetite or eating^a^	12 (85.7%)	19 (63.3%)	8 (57.1%)	39 (67.2%)	.220
Overeating^a^	9 (64.3%)	8 (26.7%)	2 (14.3%)	19 (32.8%)	.011*
Reduced food intake^a^	1 (7.1%)	5 (16.7%)	2 (14.3%)	8 (13.8%)	.694
Food preference changes^a^	6 (42.9%)	9 (30.0%)	6 (42.9%)	21 (36.2%)	.596
Onset age of eating changes^b^	64.3 ± 9.4	73.2 ± 6.7	62.3 ± 9.7	68.2 ± 9.4	.003*
Onset age of initial disease symptoms^b^	62.8 ± 8.1	70.0 ± 6.3	62.8 ± 9.3	66.5 ± 8.3	.003*
Interval between onset of initial disease symptoms and emergence of eating changes^b^	2.2 ± 2.2	2.4 ± 1.7	2.9 ± 3.5	2.4 ± 2.3	.793
Symptom duration at first assessment^b^	2.6 ± 2.3	2.4 ± 1.8	3.6 ± 4.1	2.7 ± 2.6	.334
MMSE^b^	20.6 ± 7.9	22.5 ± 5.9	21.4 ± 5.9	21.8 ± 6.3	.666
CDR global score^b^	.93 ± .62	.53 ± .30	.77 ± .86	.69 ± .57	.085

*Note*: Missing data: MMSE (bvFTD: *n* = 2, PNFA: *n* = 2, SD: *n* = 1), CDR (PNFA: *n* = 1, SD: *n* = 1).

Abbreviations: bvFTD, behavioural variant frontotemporal dementia; CDR, clinical dementia rating; MMSE, mini‐mental state examination; PNFA, progressive nonfluent aphasia; SD, semantic dementia.

^a^ Values are presented as *n* (%), *p*‐values were calculated using the chi‐square test.

^b^ Values are presented as mean ± standard deviation; *p*‐values were calculated using one‐way ANOVA.

* Significant omnibus differences (*p* < .05). Post hoc group differences: Overeating (bvFTD > PNFA, SD); onset age of eating changes (PNFA > SD, bvFTD); onset age of initial disease symptoms (PNFA > SD, bvFTD).

The mean intervals between the onset of initial disease symptoms and emergence of appetite or eating changes were 2.2 ± 2.2 years for the bvFTD group, 2.4 ± 1.7 years for the PNFA group and 2.9 ± 3.5 years for the SD group. Although the cumulative incidence of appetite and eating changes was higher in the bvFTD group, the difference among the three groups was not statistically significant (log‐rank test, *p* = .054; Figure 1b).

## DISCUSSION

In this study, we clarified the prevalence and temporal onset of eating behaviour changes across the frontotemporal dementia spectrum. Our primary finding is that, while altered eating behaviours are a common feature of bvFTD, PNFA and SD (Jiskoot et al., 2023; Park et al., 2017; Rohrer & Warren, 2010; Rosen et al., 2006), the specific symptom profiles differ. Consistent with the previous literature (van Langenhove et al., 2016), overeating was significantly more prevalent in bvFTD, distinguishing it from the aphasic variants. By contrast, reduced food intake and food preference changes occurred non‐specifically across all three variants. Although current diagnostic criteria primarily emphasise these behaviours in bvFTD (Rascovsky et al., 2011), our results suggest that clinicians should actively screen for eating disturbances in patients presenting with primary progressive aphasia (Gorno‐Tempini et al., 2011).

From a longitudinal perspective, our Kaplan–Meier analysis indicated that for the subset of patients who do develop eating behaviour changes, these symptoms can emerge within the first few years (averaging 2–3 years from initial symptom onset), across all subtypes. Given that the majority of PPA patients (>60%) remain free of these symptoms at the 3‐year mark, clinicians should provide balanced anticipatory guidance, informing caregivers that while not all patients will experience these symptoms, eating changes may emerge relatively early in the disease course for a substantial subset of patients, regardless of the behavioural or aphasic variants.

While our overall prevalence data confirm that eating behaviour changes are common across the FTD spectrum, the pattern of prevalence (bvFTD > PNFA, SD) diverges slightly from previous reports (bvFTD > SD > PNFA) (Foxe et al., 2022; van Langenhove et al., 2016). In our cohort, eating changes in SD were less prevalent than PNFA. These differences in results may stem from variations in the methods of inquiry or differences in disease stage. Our binary scoring method that determines whether all symptoms are present or absent without threshold could capture milder eating disorders in PNFA more readily. Additionally, our cohort's relatively shorter disease duration at assessment or the small sample size of the SD group (*n* = 14) may have limited the quantitative evaluation of eating changes in SD.

Behavioural and neuropsychiatric alterations in eating habits sometimes fall outside the typical scope of cognitive assessments. Because prominent language deficits often dominate clinical attention in PNFA and SD, these associated behavioural changes can be easily overlooked. Therefore, intentional screening by physicians is clinically crucial and complements allied health interventions. Furthermore, identifying specific features like overeating (a distinct marker for bvFTD) provides critical clues about potential phenotypic shifts or disease progression. Proactive screening also enables practical interventions; for instance, identifying overeating can prompt caregivers to restrict food access, whereas stereotypy may require different management strategies. Acknowledging these behavioural shifts as disease symptoms can also partially alleviate caregiver distress by providing clarity and anticipatory understanding.

Some limitations should be noted. First, the retrospective design relied on informant recall for symptom onset, which may introduce bias. Second, the relatively small sample size limits the generalisability of our findings. Third, as this study was conducted at a single tertiary centre, our cohort may not fully capture the broad clinical spectrum and heterogeneity inherent in the frontotemporal dementia spectrum. Fourth, we treated symptom presence as binary data without factoring in severity or caregiver distress scores, which limited our ability to evaluate the clinical impact of these behaviours. Fifth, our SD cohort consisted exclusively of left‐predominant cases. Given the recent recognition of right‐sided SD and its strong association with behavioural disturbances, future studies require consensus criteria to distinguish right SD from left (typical) SD and bvFTD to evaluate these profiles accurately. Sixth, we did not compare the present data with other related disorders, such as progressive supranuclear palsy or corticobasal syndrome. Demonstrating distinct eating profiles across these related syndromes will be highly valuable for differential diagnosis in future research. Finally, our cohort lacked pathological or biomarker confirmation. Future prospective studies with larger cohorts and biomarker validation are essential for elucidating the neural correlates underlying these shared and distinct eating behaviours.

In conclusion, this study highlights that altered eating behaviours are not exclusive to bvFTD but affect the entire frontotemporal dementia spectrum with an early onset. Although overeating specifically characterises bvFTD, clinicians should actively monitor for eating changes in patients with PNFA or SD.

## AUTHOR CONTRIBUTIONS

**Yuki Sato:** Formal analysis; investigation; writing – original draft; visualization. **Hitomi Hayashi:** Investigation; resources. **Kazuo Kakinuma:** Methodology; software; formal analysis; investigation; data curation; writing – original draft; funding acquisition; visualization. **Chifumi Iseki:** Conceptualization; investigation; data curation. **Shoko Ota:** Investigation; resources; funding acquisition. **Kazuto Katsuse:** Investigation; resources. **Shiho Matsubara:** Investigation; resources. **Nobuko Kawakami:** Investigation; resources. **Shigenori Kanno:** Investigation; resources; funding acquisition. **Keisuke Morihara:** Investigation; resources. **Yoshiyuki Nishio:** Conceptualization; investigation; resources; validation; data curation; writing – review and editing; supervision. **Kyoko Suzuki:** Validation; investigation; resources; data curation; writing – review and editing; supervision; project administration; funding acquisition.

## CONFLICT OF INTEREST STATEMENT

The authors have no conflict of interest concerning this paper.

## ETHICS STATEMENT

Written informed consent regarding the use of their medical information for research was obtained from all participants. This study was conducted in accordance with the Declaration of Helsinki and approved by the Tohoku University Ethics Committee (approval numbers: 2010‐505 and 2020‐1‐285).

## Supporting information


Data S1.

## Data Availability

The data that support the findings of this study are available in the Supporting Information of this article. The raw data are not publicly available due to privacy and ethical restrictions.
